# Supporting evidence-based analysis for modified risk tobacco products through a toxicology data-sharing infrastructure

**DOI:** 10.12688/f1000research.10493.2

**Published:** 2017-09-05

**Authors:** Stéphanie Boué, Thomas Exner, Samik Ghosh, Vincenzo Belcastro, Joh Dokler, David Page, Akash Boda, Filipe Bonjour, Barry Hardy, Patrick Vanscheeuwijck, Julia Hoeng, Manuel Peitsch

**Affiliations:** 1 PMI R&D, Philip Morris Products S.A., Neuchâtel, Switzerland; 2 Douglas Connect GmbH, Zeiningen, Switzerland; 3 SBX Corporation, Tokyo, Japan

**Keywords:** Systems toxicology, Data sharing, Harm reduction, Open data, Database, Website

## Abstract

The US FDA defines modified risk tobacco products (MRTPs) as products that aim to reduce harm or the risk of tobacco-related disease associated with commercially marketed tobacco products.  Establishing a product’s potential as an MRTP requires scientific substantiation including toxicity studies and measures of disease risk relative to those of cigarette smoking.  Best practices encourage verification of the data from such studies through sharing and open standards. Building on the experience gained from the OpenTox project, a proof-of-concept database and website ( INTERVALS) has been developed to share results from both *in vivo* inhalation studies and *in vitro* studies conducted by Philip Morris International R&D to assess candidate MRTPs. As datasets are often generated by diverse methods and standards, they need to be traceable, curated, and the methods used well described so that knowledge can be gained using data science principles and tools. The data-management framework described here accounts for the latest standards of data sharing and research reproducibility. Curated data and methods descriptions have been prepared in ISA-Tab format and stored in a database accessible via a search portal on the INTERVALS website. The portal allows users to browse the data by study or mechanism (e.g., inflammation, oxidative stress) and obtain information relevant to study design, methods, and the most important results. Given the successful development of the initial infrastructure, the goal is to grow this initiative and establish a public repository for 21 ^st^-century preclinical systems toxicology MRTP assessment data and results that supports open data principles.

## Introduction

### Harm reduction and modified risk tobacco products (MRTPs)

Smoking is addictive and causes a number of serious diseases, including cardiovascular disease (heart disease), lung cancer, and chronic obstructive pulmonary disease (emphysema, chronic bronchitis) ^1^. In addition to initiatives encouraging prevention and cessation of smoking, harm reduction for smokers may be achieved through the development of novel tobacco products that have the potential to reduce the risk of harm compared to continued cigarette smoking.

The U.S. Family Smoking Prevention and Tobacco Control Act defines a modified risk tobacco product (MRTP) as any that is ‘sold or distributed to reduce the harm or risk of tobacco-related disease associated with commercially marketed tobacco products’ ^2^.

Philip Morris International (PMI) is developing a portfolio of potential MRTPs to address a wide range of adult smokers’ preferences, preserving as much of the possible taste, sensory experience, nicotine delivery profile, and ritual characteristics of cigarettes, while significantly reducing or eliminating the formation of harmful and potentially harmful constituents (HPHC) ^3^. Nonclinical and clinical studies are conducted to assess the risk associated to those products and the full set of data from the relevant scientific studies will be evaluated to determine whether they substantiate reduced exposure or risk.

Insofar as nonclinical laboratory studies (safety and toxicity studies) may provide evidence regarding the relative toxicities of MRTPs, it is proposed that they are to be carried out according to a quality management system (proposed GLP Quality System ^4^). Building quality into study planning, using methods that have been validated, executed by trained personnel in adequate facilities, and with proper data management and processing practices are the essential components of such a system and are a first step in ensuring data quality, reproducibility and reliability.

### Transparency and verification in science

The adoption of MRTPs, and thereby their potential public health benefits depend on product acceptance among existing adult smokers and their actual performance in terms of reduction in risk compared to continued smoking, which in turn shall be based on robust and multi-disciplinary scientific substantiation. Ensuring that the underlying evidence and results are openly shared, in a similar way as has been proposed by the European Food Safety Authority for example ^5^, can encourage replication of the studies and increase confidence in the findings. Indeed, several studies have shown that much peer-reviewed scientific literature is difficult to reproduce for reasons such as inadequate documentation of methods and datasets and insufficient sharing of data and methods with the community ^6– 11^. Concerns on reproducibility of science have led to recent calls for a shift to better practices ^12, 13^. For example, not only is it crucial that the science is right, i.e. ensuring that the study is blinded, that experiments are repeated, reagents validated, and the analyses appropriate, but it is also important that all results are shown, including negative and positive controls. Equally, for any scientific result on MRTPs it is important that a consistent, science-based regulatory framework is used for identification of innovative alternative products that could significantly reduce the risk of tobacco related disease and death caused by cigarette smoking ^14^. Processes that encourage transparent sharing of data in a way that allows easy review and understanding will facilitate objective evaluation of the evidence.

To complement the classical peer-review system in the evaluation of the scientific evidence, several initiatives, such as CASP ^15^, BioCreAtIvE ^16^, DREAM ^17^, and sbv IMPROVER ^18^ leverage the scientific community to verify methods of protein structure prediction, information extraction, gene network inference, and systems biology, respectively ^19^. In order for the crowd to be able to review methods and/or data, it is important to prepare those in a form that is easily understandable and usable, and to collect all of the relevant information in one place. Therefore, we developed a database and associated webportal to collect relevant information on studies assessing candidate MRTPs.

### Systems toxicology

The emerging field of systems toxicology aims to develop integrated frameworks for the prediction and quantification of substance-related toxicity. Systems toxicology is broader than a simple attempt to understand the impacts of exposure at a pathway level; it is an interdisciplinary, integrated approach that depends on data produced by rapidly developing omics technologies, such as transcriptomics, metabolomics, and proteomics ^20^, which complement more traditional toxicity endpoints. The objective is to generate more comprehensive impact overviews by combining complex biological network models with quantitative measurements of impacted pathways at all levels of biochemical and biological organization ^21, 22^ to facilitate better-informed decision making as compared with traditional safety assessment alone.

The National Research Council, commissioned by the US EPA, developed a vision for 21 ^st^-century toxicity testing ^23^ characterized by a shift in focus away from traditional toxicity testing and toward the exploration of human signaling pathways whose perturbation by biologically active substances or their metabolites causes adverse health effects ^24, 25^.

Quantitative systems toxicology involves mining omics data and functional endpoints for identification of potential adverse outcome pathways (AOPs) and their component events and event relationships. AOPs, as defined by the OECD ^26^, are simplified pragmatic frameworks, which are linear in nature and connect a single molecular initiating event (MIE) to a single adverse outcome (AO) by means of non-branching, and directional sequences of key events (KEs). Supporting evidence for AOPs is arranged according to three levels of information, namely “Biological Plausibility” of the KEs (most important), “Biological Essentiality” of the KEs and “Quantitative Evidence” of the KERs (least important). Building and quantifying AOPs requires multiscale integration of all available and relevant datasets, mining of supporting knowledge, and predictive algorithms that quantify AOPs and their evolutionary and genetic diversity ^27^.

**Figure 1.**
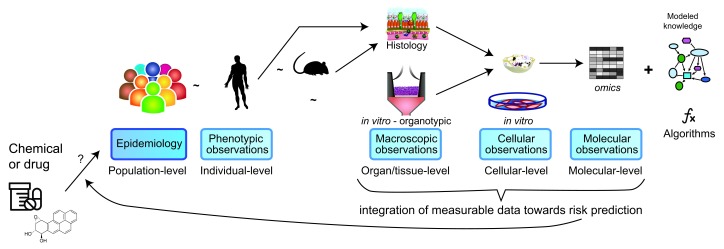
Systems Toxicology. To understand the effect and mode of action of chemicals or drugs on Human, different studies can be conducted. Epidemiology will provide the final evidence but requires long periods of observation. Phenotypic observations may be obtained at the individual level from biopsies or tissue collection. Animal studies can provide surrogate information in a controlled setup and allow the collection of various tissues and fluids. Alternatively, new *in vitro* methods are developed to provide information on toxicity and pathways of toxicity. It is possible to obtain organ-tissue level information from macroscopic observation of tissues, but also to understand cellular level or even molecular level by mining data from –omics profilings using modeled knowledge and dedicated algorithms.

Parameters that facilitate reliable quantitative prediction of toxicity and risk are also required. Multicellular and tissue simulation modeling can predict injury and repair of the tissue architecture and are parameterized by molecular models and biological assays ( Figure 1). Such a systems toxicology approach has been used successfully in *in vitro* and *in vivo* studies ^28– 32^ to assess prototypic MRTPs in the context of an integrated scientific assessment program ^3^.

### 21 ^st ^century toxicology programs and public resources

Predictive toxicology (i.e., 21 ^st^ century toxicology) is an active field that is transitioning to a mechanistic, evidence-driven science. Large international programs (e.g., Tox21 and ToxCast in the United States, EU-ToxRisk and SEURAT-1 in Europe, and TGGates in Japan – for more information, please refer to Table 1) aim to develop new biological methods and generate large datasets to probe pathways and mechanisms of toxicity that are relevant to human and environmental health. These endeavors generate increasingly complex datasets for the scientific community to analyze in the development of new hypotheses, predictive models, and integrated testing strategies. These datasets, which encompass multi-omics data, *in vitro*/ *in vivo* assays, and *in silico* toxicity prediction and modeling applied to environmental and human hazardous substances, are organized into diverse repositories (a noncomprehensive list of which is given in Table 1). Many large parent database portals and projects host or link to child databases that are available to toxicologists, regulatory agencies (such as the EPA and FDA), and the general scientific community. In addition to these initiatives, a plethora of specialized databases (e.g., ChEBI, OCHEM, and PubChem – for more information, please refer to Table 1) cover individual topics from properties of chemical compounds to biochemical assays that assess physicochemical properties.

**Table 1.** Sources of information on toxicology: database repositories for predictive systems toxicology investigations and risk assessment.

Toxicology Databases				
Database Portals/Projects				
Inh	Portal/Project name	Description	Data type	URL
	Safety Evaluation Ultimately Replacing Animal Testing (SEURAT -1)	• Cluster of 7 projects and portal to 5 databases: SCR&Tox, HeMiBio, DETECTIVE, COSMOS, NOTOX, ToxBank, and COACH • Aims to simulate repeated dosage toxicity testing in a complex physiologic but animal-free *in vitro* model • Elucidation of biomarkers and analysis in stem cell derived organotypic systems • *In silico* simulations to predict toxicity, tissue dosimetry, and proof of concept that new strategy accurately predicts liver toxicity *in vivo*	Access regulated per dataset; no inhalation data	http://www.seurat-1.eu/
	BioSharing	• Portal of curated web-based, user- queryable registries of linked information on content standards, databases, and data policies in the life sciences; broadly covers the biological, natural, and biomedical sciences	Several databases	https://biosharing.org/biodbcore/
	EU-ToxRisk	• Integrated European program aiming towards mechanism-based toxicity testing and risk assessment in nonanimal organotypic human *in vitro* models • Applies mechanistic molecular knowledge to develop ‘Adverse Outcome Pathways,’ enabling *in silico* knowledge generation regarding toxicity • Integrates information from cellular and molecular biology, computational toxicology models, and systems biology to assess toxic responses to repeated chemical exposure	Website under construction	http://www.eu-toxrisk.eu/
	Data Infrastructure for Chemical Safety (diXa)	• Collection of European toxicogenomics experiments with crosslinks to several globally-available chemical and molecular medicine databases • Contains data across several disciplines, including chemical toxicity, dosimetry, omics analyses, and chemical catalogues, integrated into a single resource with a focus towards the development of nonanimal tests for prediction of chemical safety	Individual cigarette smoke (CS) chemical information	http://wwwdev.ebi.ac.uk/fg/dixa/ index.html
	TOXNET	• Collection of several databases and publications covering topics such as chemicals and drugs, diseases and the environment, environmental health, occupational safety and health, poisoning, risk assessment and regulations, and toxicology • Provides links to PubMed and NLM interface for associated publications in biomedical toxicology field	Mostly publication abstracts and links	http://toxnet.nlm.nih.gov/
	ToxCast™/Tox21	• Tox21 is a United States federal collaboration to develop better toxicity assessment methods. Tox21 has resulted in the screening of over 10,000 chemicals via ~50 high-throughput assays • The EPA has contributed the chemical screening results from the Toxicity Forecaster (i.e., ToxCast™), an initiative to assess and screen ~2,000 chemicals for toxicity to cells and proteins via over 700 automated, high-throughput screening assays • Data consist of chemicals used, assays performed, genes and pathways implicated, and endpoints	Data available via iCSS dashboard: individual CS chemical information, publication links, graphs, and assay data files	http://actor.epa.gov/dashboard/
Inh	Individual Databases			
	PubChem	• Provides information on small-molecule biochemical activity • Specialized databases within PubChem (PCSubstance, PCCompound, PCBioAssay) contain physical and chemical properties and nomenclature of over 100 million substances • Cross-linked entries across the three databases (Substance, Compound, and BioAssay) and NCBI Entrez • PCBioAssay database contains bioactivity screen information, raw assay data, and readouts of screening protocols for chemical substances in PCSubstance	Publications and assay records	https://pubchem.ncbi.nlm.nih.gov/
	ChEBI	• Dictionary of identifiable, distinct molecular entities (nucleic acids, peptides and proteins) linking various classes of entities and their parents/ children • Follows the nomenclature and terminology laid out by IUPAC and NC- IUPAC • The ontology classes encompass various science and engineering disciplines (e.g., nicotine is linked to its structural, chemical, and biological properties) • Provides links to several other databases and publications for chemicals	Individual CS constituents, disease associations	https://www.ebi.ac.uk/chebi
	Comparative Toxicogenomics Database (CTD)	• Collection of interlinked public databases with information on the effects of drugs and chemical exposure on human biochemical processes • Manually curated information from scientific journal articles on chemical- disease and gene-disease relationships integrated with various other data, including those of pathways (e.g., KEGG) and gene ontology, to elucidate the underlying molecular landscape of environmentally borne diseases	Independent studies and publications	http://ctdbase.org/
	Aggregated Computational Toxicology Resource (ACToR)	• Publicly available online resource for toxicity data for over 500,000 chemicals accumulated and referenced from EPA repository of toxicity databases such as ToxCastDB (chemical screening data), Exposure Data (effects of chemical exposure on humans), and DSSTox (structural and annotation information) • Provides physicochemical, *in vitro* and *in vivo* toxicology data on many toxic substances, including industrial chemicals, pesticides, and other contaminants	Publications, reports/ surveys from other government/private agencies, and other databases and studies	http://actor.epa.gov/
	Chemical Effects in Biological Systems (CEBS)	• Toxicogenomics database with a conglomeration of omics, classical toxicology, gene, and protein regulatory network data on human health and environmental toxicology • User-queryable interface allows search for protocols, chemicals, endpoints, genes/proteins, tissue type, toxicological parameters, etc., to facilitate hypothesis- driven systems toxicology research and risk-assessment studies	Publications, reports/studies from government/ private agencies, databases, and independent investigations *Links ToxCast21 Phase II	https://www.niehs.nih.gov/ research/resources/databases/ cebs
	CompTox Chemistry Dashboard	• Access to >740,000 chemical substances associated with both experimental and predicted properties		https://comptox.epa.gov
	Online Chemical Database (OCHEM)	• Modeling tool for development of substance properties-quantitative structure-activity and structure-activity (QSPR/QSAR) models • Repository for scientists’ models to allow cross examination and estimation of the ADMET properties of any compound		https://ochem.eu
	ToxBank	• Subsidiary of the SEURAT-1 project that unifies all the *in vitro*, *in vivo*, and *in silico* data and experimental protocols under one roof to facilitate integrated data analysis • Enables the development of an industry standard data repository to replace repeated *in vivo* repeated dose toxicity testing • Compound wiki provides information on selected hepato-/cardiotoxic compounds	Access is regulated per dataset; no inhalation data	http://toxbank.net/

The table highlights sources of information on *in vivo* chemical inhalation and individual *in vitro* chemical toxicity. The type of data available and, where known, user accessibility (e.g., open source vs licensing) have also been highlighted. While a number of databases and portals are still active, a few of them are no longer maintained. Green color in the “Inh” column means that the resource contains inhalation data.

For example, OpenTox ( www.opentox.net) ^33^ was started as a project of the European Commission’s Seventh Framework Program: HEALTH-2007-1.3-3; it compiles specifications, standards, and tools for the integration of data, algorithms, and models from various public and confidential toxicological sources. It was designed as an open framework for the generation and validation of computer models of toxic effects, libraries for the development and seamless integration of new algorithms, and scientifically sound validation routines. After the end of the initial R&D project in 2011, OpenTox evolved into a practical community resource, extending to all aspects of risk assessment, including experimental design, data management, biological data analysis, modeling, AOP development, and regulatory issues. Finally, this resulted in the foundation of the OpenTox Association ( http://www.opentox.net/the-opentox-association) in 2015. This initiative integrates knowledge, processes, and people from many different fields, including toxicology, biology, chemistry, bio- and cheminformatics, and computer science, by organizing community interactions (e.g., working groups, workshops, scientific meetings, and hackathons in the United States, Europe, and Asia). Additionally, OpenTox members are involved in major research projects, such as SEURAT-1: Towards the Replacement of *in vivo* Repeated Dose Systemic Toxicology Testing ( www.seurat-1.eu) and its follow-up project EU-ToxRisk ( www.eu-toxrisk.eu).

To facilitate better-informed decision making in risk assessment, knowledge integration may include evidence from *in vivo*, *in vitro* or *in silico* methods; biology, chemistry, or engineering; and human-health- or environment-oriented research. There are growing opportunities to base knowledge integration and sharing on a combination of emerging concepts and frameworks. Such frameworks require a clearly defined ontological and knowledge basis, and all applications need to employ sound, reproducible scientific methods and good practices in terms of experimental characterization, data organization, and concept description ^34^. One challenge in knowledge integration is that in many areas of predictive toxicology and safety assessment, scientific knowledge is generated not only by existing methods accepted by regulators, but also by a growing number of alternative research methods and initiatives, for which the data and their structures may be less well defined. Hence, as indicated by Gary Miller in his editorial, “Data Sharing in Toxicology: Beyond Show and Tell” ^35^, the quality of the necessary infrastructure for harmonized data sharing has lagged far behind that of the actual data. To facilitate verification of research conclusions, data need to be organized and managed carefully and traceably, processed with a variety of workflows and analysis techniques, and shared with the community for scrutiny and further analysis so that they ultimately generate knowledge. Therefore, data integration, meta-analysis, and the interaction of data and predictive models with existing knowledge frameworks (e.g., AOPs describing the sequence of key events leading to stress, repair, or toxicity) are becoming increasingly important.

While several public data resources, as identified in this section, have been developed to provide systematic access to multidimensional systems toxicology data, the ever-increasing disaggregation of data, information, and publications throughout various channels (e.g., blogs, public health news, journals, and key opinion leaders in specific fields) make it challenging for researchers to filter, pursue, and focus on relevant knowledge sources. Thus, a single cloud-based dashboard that aggregates, assimilates, mines, and prioritizes data and information according to relevance could play a central role in enabling an open, data-driven, evidence-based platform for 21 ^st^-century toxicology studies. Such a tool may also facilitate identification of key opinion leaders and experts who could perform in-depth reviews of specific data and/or results.

Despite the availability of much information in fields related to systems toxicology, few databases provide integrated toxicological evidence for respiratory analysis/assessment (e.g., for study of *in vivo* chemical inhalation). Databases such as ACToR ^36^, the Comparative Toxicogenomics Database (CTD) ^37^, and CEBS ^38^ do contain some independent studies focused on inhalation-associated chemical toxicities, but presently, corporations and others interested in inhalation toxicology, who are focused on assessment and mitigation of toxicity associated with inhalation of substance constituents, have no central “go-to” repository. Therefore, approaches to utilize already-present data in reproducible analyses or extract relevant conclusions for specific investigations have been limited by poor coordination and crosslinking and the lack of integrated, harmonized, open access and availability of data.

Collaborative aspects of the systems toxicology approach can be founded on projects such as sbv IMPROVER ^18^, which verifies techniques in computational biology using crowd sourcing to facilitate analysis and understanding of large, complex datasets.

In this paper, we describe emerging data practices we have developed to support a robust, reproducible predictive toxicology/safety assessment applicable to inhalation science in the context of novel and alternative tobacco products. We also describe a proof-of-concept implementation of a data-sharing infrastructure as the underlying foundation of a knowledge-sharing portal on novel tobacco and alternative products. Here we do not focus on the quality framework in which studies are performed, but emphasis is placed on sharing of information on protocols, and raw and processed data in a standardized way.

## Methods

### INTERVALS: Inhalation toxicology repository for MRTPs

**Figure 2.**
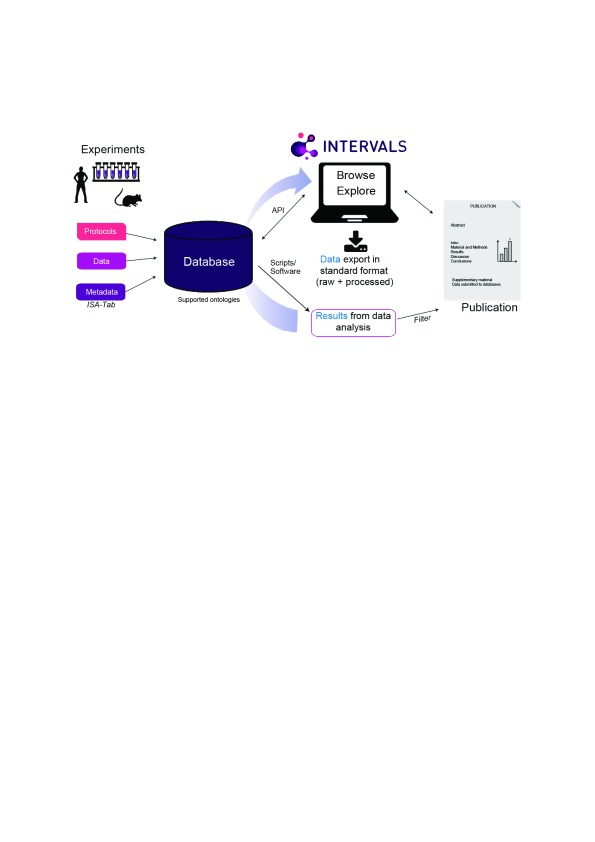
Concepts of infrastructure and data sharing. Ideally, as experiments are performed, protocols and metadata are recorded for each of the data entries and curated in ISA-Tab files. They all are imported into a common database that supports defined ontologies. Raw data can be exported from this database and processed with different scripts and/or software to generate analyses results, some of which are usually shared in a publication. All of the results can be saved into the database and the data and results can be accessed through an API to be browsed on and downloaded from the website named INTERVALS. The website also keeps track of publications associated with the studies.

A database and searchable web portal (INTERVALS) have been developed as proof-of-concept for data sharing in systems toxicology. They include results from *in vivo* inhalation studies and *in vitro* studies conducted by PMI R&D to assess candidate MRTPs ( Figure 2). The website and underlying database can be accessed at www.intervals.science and should allow the scientific community to easily retrieve relevant and usable information relevant to MRTPs from a single place and with similar standards (described below).

The data modeling described below adopted the latest standards of data sharing and reproducible research. Therefore, the INTERVALS vision underscores the importance of a central repository for toxicological inhalation data and encourages sharing of information and expertise across the scientific and regulatory communities to foster reproducibility in predictive toxicology and risk assessment.

The workflow development and principles of data preparation and database infrastructure largely reused open computing resources and standards developed within OpenTox, both for designed programs and associated community engagement. Particularly, OpenTox’s engineering design as an interoperable distributed framework of components, that interact via well-defined application programming interfaces (APIs) and web services, provided a strong technical base for the extensible integration of diverse software components and resources. OpenTox includes services for data integration, model development, validation, and reporting that can satisfy scientific, community, and regulatory requirements for sustainable extension of data management, validation, and regulation.

Another integral part of the portal’s vision is assimilation and mining of data for identification of scientifically relevant information and identification of key experts to facilitate and validate reviews and analytics. In this paper, we focus on the data science practices developed to support verification of conclusions derived from systems toxicology studies, illustrated by a case study example.

### Studies and datasets

For proof-of-concept, a number of datasets from assessment studies conducted by PMI R&D on prototype or candidate MRTPs were prepared and integrated into the platform. Two examples are detailed below to exemplify the new data-management and sharing philosophy for large *in vivo* and *in vitro* studies. To learn more about these studies’ results, please refer to the respective publications, as only short descriptions are given below.

1) Chronic obstructive pulmonary disease (COPD) progression in response to chronic exposure to cigarette smoke (CS) or a prototype MRTP (pMRTP) (i.e., the C57BL6-pMRTP-SW dataset) ^31^. Cigarette smoking is a cause of COPD. Thus in the assessment of MRTPs it is of interest to understand to what extent the risk of COPD may be reduced in comparison to exposure to cigarette smoke. Using a systems toxicology approach in a model of COPD (C57BL/6 mice), the potential of such a pMRTP to reduce health risk was assessed. The study investigated physiological endpoints in parallel with the transcriptomics, lipidomics, and proteomics profiles of mice exposed to CS from a reference cigarette (3R4F) or a pMRTP aerosol for up to 7 months. In addition to the control (fresh air-exposed) group, the study also included a cessation group and one that switched to the pMRTP after 2 months of 3R4F exposure to evaluate the potential risk reduction of switching to pMRTP compared with continuous 3R4F exposure; those results were benchmarked to cessation.
2) *In vitro* assessment of the effects of acute exposure to the aerosol of a candidate MRTP, the Tobacco Heating System version 2.2 (THS2.2), on human three-dimensional (3-D) organotypic buccal or nasal tissue cultures (i.e., the organotypic buccal and nasal datasets, respectively) ^39, 40^. The recently developed 3D organotypic buccal and nasal epithelial culture models offer physiologically robust systems to study the effects of inhalation exposure. Biological impacts were assessed following exposure to aerosol generated from THS2.2 as compared with CS from reference cigarette 3R4F. The experiments were repeated with multiple applications of the aerosol or CS to obtain reproducible measurements or reliable observations of molecular and cellular changes following exposure. Aligned with the 3Rs strategy (i.e., replacement, refinement, and reduction) and the Vision and Strategy of Toxicity Testing in the 21st Century ^41^, a systems toxicology approach found that at all tested concentrations, 3R4F CS had considerably greater impacts than THS2.2 aerosol in terms of cytotoxicity, tissue morphological alterations, secretion of proinflammatory mediators, impaired ciliary function, and perturbation of transcriptomes and miRNA expression profiles ^39, 40^.

### Protocols

We propose to follow the best practice of requiring all data uploaded to the community portal and the supporting data repository to have well-documented protocols describing the methods followed to generate and process data as developed within the ToxBank project. In the current version, summary protocols and key steps in data production and processing are included in the ISA-Tab files. Future development of the INTERVALS database and site will allow for protocol versioning. When a new protocol has been developed, documented, and reviewed, it will be uploaded to the data repository by the investigator following guidelines on the content and organization of the protocol description. The protocol will be loaded through the portal’s upload interface, where additional information associated with the protocol, including a protocol summary and identification of the protocol’s owner and authors. In addition, keywords from our supporting ontology are assigned to support the search function. The protocols will be visible and downloadable on a dedicated set of pages in INTERVALS.

### Standards

Following the recent dataset preparation work in ToxBank supporting SEURAT-1 (and its successor, the EU-ToxRisk program), a strategy proposal on data presentation was prepared and shared with the OpenTox data working group ^42^. This proposal was further expanded based on use cases and datasets from PMI and additional community inputs and experiences from other projects (e.g., ToxCast, Tox21, and TGGates). It incorporates ISA-Tab files to describe experiments, data production, processing, associated metadata, and the use of defined ontologies.

Importantly, data interoperability and submission to regulatory agencies requires conformance to strict data standards (e.g., for FDA submission, refer to the guidance for submission of electronic data ^43^). Protocols, metadata, and data files have been prepared to follow the FAIR principles (i.e. Findability, Accessability, Interoperability, and Reusability) ^44^ to the extent possible. This implied specific rules during data curation, as well as specific design for dataset retrieval in the search tool described below.

### The Investigation/Study/Assay Tab-Delimited (ISA-Tab) Standard

Sustainable dataset storage requires not only a defined data format but (even more importantly) well-organized, annotated metadata on the experimental setup. The ISA-Tab standard was created for this purpose and has already been used in projects like the ToxBank ^45^ infrastructure of SEURAT-1 ( www.toxbank.net) and diXa ^46^ ( http://www.dixa-fp7.eu/); an extended version, ISA-Tab nano, was used in the eNanoMapper project ^47^ ( www.enanomapper.net).

The ISA-Tab format ^48^ is a standardized, general-purpose framework for the collection and communication of complex metadata that consists of three types of tables: the Investigation, Study, and Assay tabs (I-, S-, and A-tabs, respectively). The I-tab summarizes general information on the complete investigation, all studies, and all assays, including people involved in the investigation, related publications, and short protocol descriptions. Additionally, it relates the A-tabs to the S-tabs. The S-tab contains information on the study subjects, their characteristics, and any treatments applied. Finally, the A-tab describes the smallest complete unit of experimentation that produces data associated with a subject.

The ISA-Tab specification has a somewhat different definition of study and assay compared with their use in normal lab settings: an ISA-Tab investigation corresponds to a complete experimental design, often called the study design in practice. Under the ISA-Tab specification, a study deals with the *in vivo* or *in vitro* sample and an experimental assay conducted to investigate a specific endpoint, such as transcriptomics. To circumvent possible confusion caused by this contradictory use, we place “ISA-Tab” in front of the terms “study” and “assay” if they are used according to the ISA-Tab definition. The advantage of ISA-Tab is that its generality imparts the flexibility to provide metadata for almost any experimental setup. However, ISA-Tab files from different groups or projects might look very different, even if all files are consistent with the ISA-Tab standard, because of the metadata’s undefined structure. This applies not only to the specific metadata included in the files but also to the splitting between the S- and A-tabs.

Initiatives such as the ToxBank project have attempted to standardize the ISA-Tab format for toxicological applications (i.e., ISA-Tab investigations). So far, the focus has been on relatively small studies with only one or a few endpoints, and the files have been created after study completion, usually by people involved only in parts of the study (or not directly involved at all). For studies like the examples here, which have more complex designs, including multiple tissues and endpoints, this approach is complex, time-consuming, and error-prone, requiring the ISA-Tab files’ creator to consolidate experimenter input and validate the files. Therefore, herein, we propose a new ISA-Tab scheme that follows the data-production workflow and combines data production and documentation into a single step by the researcher, who is the expert on the dataset and experimental parameters.

**Figure 3.**
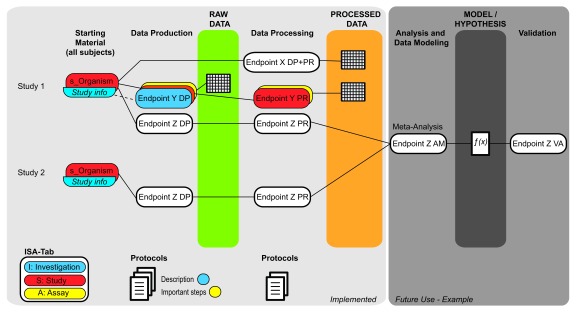
Schematic of the hierarchical structure of interconnected ISA-Tab instances. The schema depicts the theoretical splitting strategy of data and metadata from two different studies into ISA-Tab files. The highest level will describe all subjects or samples analyzed in a study. Then, for each endpoint, a file describes the data production step, and links out to a raw data file. Another file will describe data processing steps and link out to processed data files. It is also possible that the two steps are combined into a single file. Eventually, analysis and data modelling could consider data from multiple studies.

Instead of one ISA-Tab instance documenting the complete study, a hierarchical structure of interlinked ISA-Tab files was created to follow the study’s experimental steps ( Figure 3). The steps covered in specific ISA-Tab instances can be handled flexibly according to tasks performed by different labs, sites or collaborators, even before the full study is completed. New endpoints can be added easily, and the files can be updated if additional information (e.g., publications) becomes available.

The resulting interconnected ISA-Tab instances were hierarchized into different levels. The highest, most upstream one is the system (SY) level, which describes the main subjects under investigation (i.e., the animals or tissue cultures and their treatment by chemicals for *in vivo* and *in vitro* studies, respectively). The next layers describe data acquisition and analysis. For endpoints with various options to derive processed data from raw data, splitting the experiment description into data production (DP) and processing (PR) sets of ISA-Tab instances documented different processing options as independent assays. Layers 4 and 5 were reserved for modeling and validation; these can combine information from different ISA-Tab instances from layers 1–3. To document interconnections between files, the upstream ISA-Tab instance is referenced in the S-tab of the downstream ISA-Tab instance. This ISA-Tab splitting approach is illustrated in Figure 3, and its applicability is demonstrated with a specific example below.

The C57BL6-pMRTP-SW and organotypic studies were imported according to the above novel concept, resulting in separate ISA-Tab instances for the different endpoints, which can then be used as templates for additional studies.

**Figure 4.**
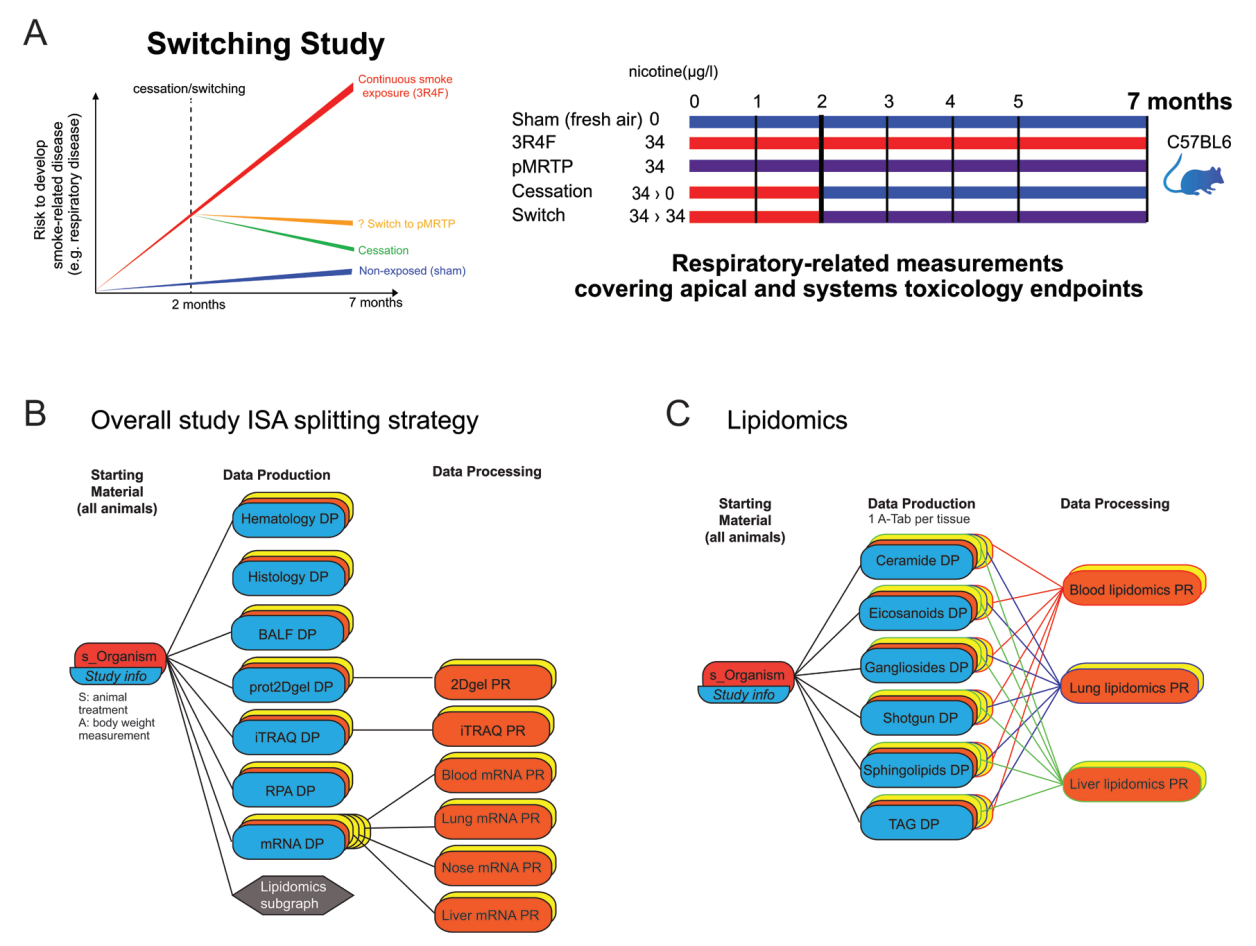
Study design and organization of the ISA-Tab instances for the C57BL6-pMRTP-SW study. **A**. Switching study concept and study design and setup. **B**. ISA-Tab splitting strategy of endpoints. The data production (DP) and processing (PR) instances describe the experimental setup and processing steps, from raw to processed data. Transcriptomics processing is separated by tissue, resulting in individual PR ISA-Tab instances. **C**. A more complicated lipidomics scheme was necessary because the experiment was performed independently for different groups of lipids, and hence separate DP instances were used for each mass spectrometry platform/set of methods. Processing was then performed per tissue, resulting in separate PR instances for blood, right lung, and liver. For simplicity, data files are not depicted here.

The complete C57BL6-pMRTP-SW study was performed on the same population of mice, which were exposed to reference CS, the aerosol from a pMRTP, or fresh air. Exposure conditions were summarized in the SY-level ISA-Tab instance, whereas body-weight measurement was covered in an A-level ISA-Tab instance. Data production for the different endpoints was described in the second level, and some endpoints had third-level instances for raw data processing. The complete structure of the ISA-Tab instances is presented in Figure 4.

In the following paragraphs, we present the requirements for specific endpoints that were considered during ISA-Tab development. Endpoints without such requirements are not listed but are included in Figure 4.

**Transcriptomics**: Gene expression was measured in four different tissues, each of which was covered as a separate A-tab in the DP instance. Because processing differed between the four tissues, four separate PR ISA-Tab instances were created.

**Lipidomics:** The exact metabolite-profiling procedure is dependent on the lipids analyzed. Therefore, one DP ISA-Tab instance was created for each group of lipids, which each had three A-tabs for the different tissues for which lipidomics data were available. Similar to the integration of transcriptomics data, the processing was done on a per-tissue basis. The PR ISA-Tab instances incorporate information from all six DP instances, all of which were accordingly referenced as upstream ISA-Tab instances.

**Proteomics:** Protein-expression profiling data were measured using three different, separately assembled experimental approaches. For 2D gel electrophoresis and iTRAQ, data preparation and processing were split between two ISA-Tab instances so that they could be used as templates for future studies, in which they could facilitate any necessary alternative processing options. Proteomics data acquired using Zeptomark reverse protein arrays were described in a single instance that combined data production and processing.

**Bronchial alveolar** **lavage fluid** **(BALF)**: Cell count measurement and multi-analyte profiling performed on BALF were treated as two A-tab assays in one DP instance.

**Histology and histomorphometry:** Histology and histomorphometry measurements were covered in two A-tab entries in the same DP instance.

**Figure 5.**
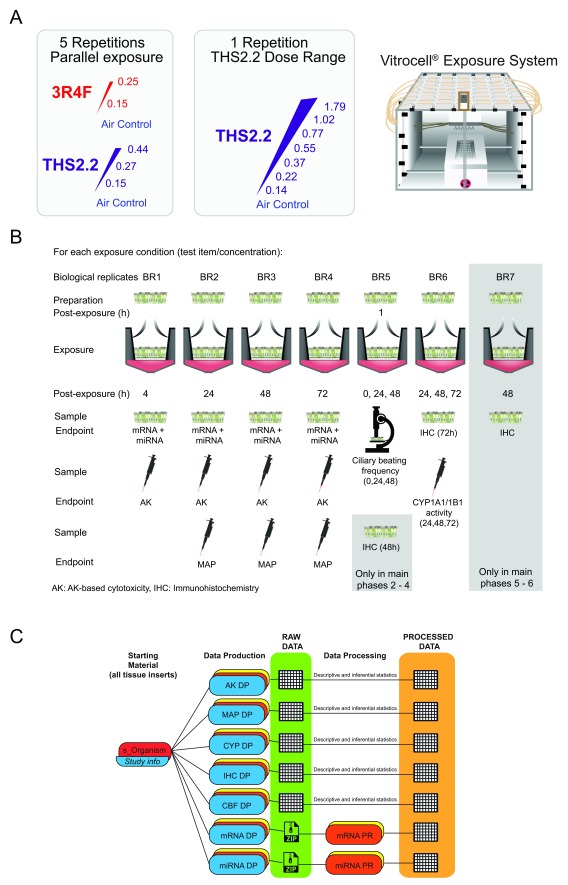
Organotypic dataset – Study design, setup, and ISA-file splitting. **A**. Study design and setup. **B**. Measurement type per insert. For each condition (test item type and concentration), a set of up to seven inserts was used to measure endpoints at different post-exposure times. Longitudinal measurements were conducted for CBF and CYP1A1/1B1 activity. For other endpoints, a new insert was used for each post-exposure time point. **C**. ISA-Tab splitting strategy of endpoints data production and processing across ISA-Tab files. Raw and Processed data files are illustrated with green and orange backgrounds, respectively.

The organotypic nasal *in vitro* study included a range of functional and molecular endpoints: adenylate kinase assay as a proxy for cytotoxicity assessment, cytochrome P450 activity, profiling of proinflammatory mediators (MAP), ciliary beating frequency measurement, histological analysis, and molecular endpoints (mRNA and miRNA). The overall study design and ISA-Tab splitting strategy are illustrated in Figure 5.

Even if the use of multiple ISA-Tab instances is convenient for data input, a central source of information on each specific endpoint for further analysis, validation, and prediction is desired. Therefore, the SY, DP, and PR instances were compressed into a single Microsoft Excel file per endpoint. Because this file format facilitates the inclusion of multiple sheets per spreadsheet file, the structure of split, interlinked ISA-Tab instances can be maintained.

### Ontologies

Templates for data and metadata for different endpoints do not necessarily define standard file formats that everyone has to follow strictly. Efforts to define such standards often face the challenge that resulting formats are not sufficiently flexible to keep up with new developments in a dramatically changing field like *in vitro*/ *in silico* toxicology and are thus limited to specific applications. For example, the SEND format ( https://www.cdisc.org/standards/foundational/send), developed by the Clinical Data Interchange Standards Consortium (CDISC) and advocated by the FDA as standard file format, was designed for regulatory reporting. However the controlled terminology included in SEND does not offer the required flexibility to support reporting of systems toxicology data. The inclusion of nonstandard data and controlled terminology therein is very complicated, and extensions to the standard require the approval of and can only be integrated by CDISC. The ISA approach tackles the harmonization and interoperability problems in another way. Even if the S- and A-tabs are only defined as momentarily required, the tabular form, content, and order of the columns can be freely chosen, and data sharing is possible, because ontologies are used in the metadata’s annotation. Users and computational tools can understand the data associated with specific entries by searching for words in this controlled terminology. Unfortunately, no ontology covers everything from sample preparation to experimental setup and endpoint readouts. While defining the ISA-Tab templates, the ontology terminology for metadata also had to be selected. This involved selections between publicly available ontologies (e.g., the ones available through BioPortal ( http://bioportal.bioontology.org)) and defining metadata without the use of ontology (e.g., additional terminology could be defined and included in a new ontology/metadata). For example, a new ontology had to be created to cover terms relevant to studies on cigarette smoke ^49^. There is no single correct decision, and as ISA-Tab is a relatively new standard, no consensus has been established on templates, optimal representation, or hierarchy; this area of emerging data science practice is supported by discussions within the OpenTox Working Group ( http://www.opentox.net/wgsmainpage). To support these activities, we provide the ontologies selected to describe the above-mentioned datasets and describe recently published ontologies of interest in Supplementary File 1. Additionally, we list three ontologies whose use is discouraged because they are less well defined than (or are amalgamations of) the other entries. In the future, this ontology collection will be extended to satisfy additional experimental needs. The development and incorporation of ontology supports the creation of a robust knowledge infrastructure to achieve semantic interoperability and the associated benefits of reliability, evidence integration, and accuracy of reasoning. A lookup service to find entries in these ontologies and automatically add the resulting terms to the ISA-Tab files during template creation is currently being developed. This tool will also be able to assign multiple ontological entries to a specific term; this is needed because of the parallel development of overlapping ontologies with slightly different words for the same object.

### Quality control and data upload

Currently, data collection and generation of the ISA-Tab files are performed manually, with rudimentary automation using Excel. To guarantee the datasets’ quality and accuracy, multiple iterations of a checking cycle involving the researcher, modelers, and data managers are conducted. Even if manual data curation will still be necessary for final quality checks, automation of this process is ongoing. Equipment data, log files, already-available databases, and computational infrastructure will be interfaced to provide the needed information at least partially. This will reduce the effort needed for quality control, because it facilitates the avoidance of copy-and-paste errors.

The first step in this direction is the development of a data upload and management application consisting of several web forms and lists with filtering/searching features connected to the uploaded datasets. The input forms are separated into two sections for management of facet terms and the dataset; both sections run in the context of the database management environment, which incorporates tools for user/access token management and access logging.

Even if the simple application does not support any validation features with respect to the correctness of the data files, it already offers the following benefits:

• centralized management of dataset information;
• prevention of problems associated with parallel work/versions;
• controlled vocabularies for facets;
• prevention of filename mismatches and other errors;
• history/log files of addition and modification; and
• automatic backups.

### Dataset search and access

**Figure 6.**
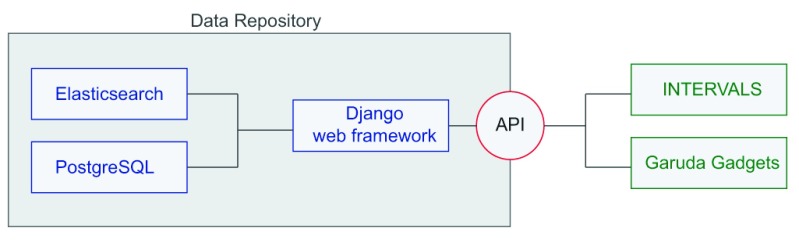
Data repository overview and links to website and tools through an API.

The data repository provides data storage and retrieval according to the OpenTox specification ( http://opentox.org/dev/apis/api-1.2). It is implemented as a client–server architecture wherein the server exposes an API to which clients connect to search and retrieve data. The data repository contains implementations of the following open source technologies (see Figure 6): Elasticsearch ( https://www.elastic.co/) as a dataset metadata store that provides search/faceting, PostgreSQL ( http://www.postgresql.org/) as a store of administrative/access data, Django framework ( https://www.djangoproject.com/) as an HTTP web server to provide dataset management, and search-request validation and processing, and JSON as the underlying data-transfer format.

**Figure 7.**
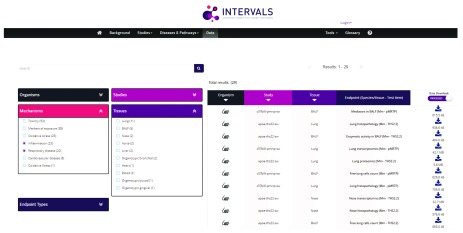
Faceted search user interface. Users can filter the datasets by Organism, Study, Mechanism, Tissue/Organ, and Endpoint Type. A toggle switch provides a choice between downloading raw and processed data.

The two OpenTox HTTP REST-compliant endpoints of the API are search and data retrieval ( Figure 7). The search endpoint has full text search (usually found in data-retrieval services) and faceted search facilities. Faceted search allows users to explore the data collection by applying multiple filters whose values are selected from predefined categories (facets) assigned to the datasets. The facets used to classify the present project’s data can be extended easily in the future, but they presently include study, study type ( *in vivo*/ *in vitro/clinical*), mechanism, exposure, organism, system, tissue, and endpoint. At each filtering step, users are presented with the number of datasets currently filtered. We determined that faceted search is an effective extension to the usual full-text search approach, as it provides users with not only data retrieval but also quick, user-friendly data exploration. The data endpoint returns requested datasets that include either raw or processed data and are enriched with additional metadata information stored in ISA-Tab files. For convenience, each dataset is served as a ZIP file that includes all the mentioned parts.

## Discussion and outlook

Within the scope of this proof-of-concept definition and implementation phase, we identified and clarified data requirements and developed a common framework for preparing relevant datasets to share with the community. The support of ISA-Tab by a data infrastructure that is interoperable with OpenTox and partner resources such as Garuda ^50^ represents a high-quality and sustainable data-science solution, extensible beyond the presently demonstrated application.

Besides data access and sharing, our goal is to present different stages of processed data, so that users can distill the raw data to strengthen their examinations. The data were prepared according to high-quality data-science methods and can be analyzed rigorously by biologists and computational biologists. Physicians and pathologists may need more refined and processed data for their consumption following methods of evidence-based medicine ^51^ that were recently extended to toxicology ^52^. Biologists can process and analyze data and publish results; those results can then be used translationally by medical scientists, who can interpret the evidence further for clinical use.

The goal for advancement of alternative testing methods (e.g., those pursued by the SEURAT-1 and EU-ToxRisk programs and supported by OpenTox and ToxBank) is the development of a stronger scientific framework for assessment of systemic toxicity, which could lead to the reduction/replacement of many expensive chronic animal experiments. To achieve this challenging goal, we need to perform case studies to integrate heterogeneous evidence from *in silico*, *in vitro*, and *in vivo* sources to support verification and validation of new methods. The preparation and sharing of dense, high-quality datasets—as described in this paper—is expected to facilitate effective review. In the following sections, we will describe additional relevant topics, which will be priorities in our further extension of the data infrastructure and webportal utilization for in-depth peer review.

### Interoperable data analytics – example of dedicated Garuda gadgets

In order to interpret large scale-omics data, filter the signal from the noise and lead to actionable insights, researchers need to focus on the "biological small data”, i.e. data which leads to meaningful information and contributes to knowledge about living systems when put into the right context. To extract meaningful information and knowledge from data, researchers need to use a diverse set of computational tools, algorithms, database and analytical services. Various computational approaches have been developed to study biological systems at the level of genes, transcripts, proteins, metabolites to cells, tissues organ and whole body modeling. Most analyses require the use of multiple databases, tools and software in different contexts, and more often than not, it is not possible to define the set of tools and their sequence of connections *a priori*. The Garuda platform is an open and community-driven platform providing a framework to connect, discover and navigate through different applications named gadgets, databases and services in biology and medicine ^50, 53^. The strength of Garuda resides in the language-agnostic build: APIs allow to connect software written in any programming language as gadgets. Moreover, the dashboard allows to explore all available gadgets through the gateway. The Garuda platform enables users to access the data from INTERVALS and to analyze and visualize it through customized gadgets on a dashboard accessible from the INTERVALS platform.

### AOP-based risk assessment

**Figure 8.**
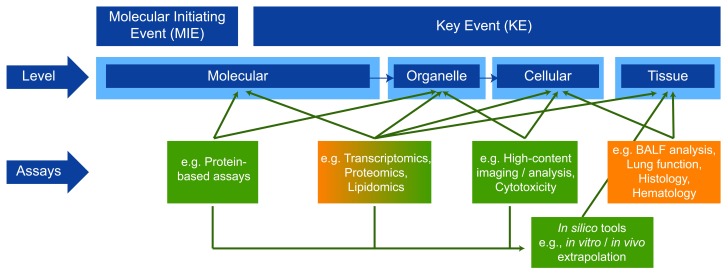
Schematic of an AOP. The schematic includes biological assays to test the molecular initiating event and specific key events on different levels, which could be combined into a weight of evidence supporting risk assessment or integrated testing strategies. The *in vivo* tests (orange) should be increasingly replaced by a combination of *in vitro* assays and *in silico* tools (green) to reduce animal testing according to the 3Rs principle ^26^.

In the near future, a combination of *in silico* methods, including toxicokinetics modeling, could be used for mechanistic extrapolation of *in vitro* data and background knowledge to human *in vivo* risk assessment according to cross-applicability and/or AOPs. For example, the strategy could integrate evidence from distributed OpenTox resources into AOPs and Risk21-based risk assessments ^54^. Starting with harmonized data that are accessible from interoperable services, such as the one described here, a variety of analyses and visualization procedures may be applied. On the basis of such analyses and the knowledge collected in AOPs ( Figure 8), the weight of evidence supporting risk assessment and integrated testing strategies could be increased, as already successfully demonstrated by Jaworska and colleagues ^42, 55^. in identification of potential skin sensitizers.

It would be particularly attractive to move between different chemical or endpoint spaces using biological signatures. Further, these methods and tools are transferable to other problems of societal concern (e.g., health/safety assessment of new products, safety biomarker discovery, air-pollution risk management, nanotechnology innovation, toxic-dust exposure, and green chemistry). Although some of these goals and activities may be challenging, we suggest that promotion of interdisciplinary data-science practices into an evidentiary framework can significantly advance the development of such alternative methods and engage support for and community involvement with the motivations of 21 ^st^-century toxicology.

### Reporting

The present infrastructure was developed with a research perspective in mind. The collected metadata in the ISA-Tab files represent the information needed to recapitulate the findings of the corresponding scientific publications and perform additional analyses. For regulatory purposes, additional and different information would be needed. For example, animals or samples excluded from analysis would need to be reported. Additionally, file formats like SEND and OECD harmonized templates (HT) require descriptions of file formats, which are not included in the ISA-Tab standard. Defined templates for data and metadata are only available for a limited number of endpoints, even though work is in progress to increase the scope of SEND format to for example *in vitro* testing. The focus of SEND files is on the data and its annotation using controlled terminology. ISA-Tab files focus more on the protocols used and metadata, and one could imagine combining both standards for a full description of the data, metadata, and protocols. Therefore, we are presently investigating extensions to the ISA-Tab templates and data infrastructure to facilitate reporting of metadata and file generation in the needed formats (whether SEND, OECD HT, or any other emerging reporting standard) on the fly.

### Intelligent system for knowledge mining and visualization

Access to high quality, curated datasets are a fundamental step towards verifiable and reproducible science. At the same time, the ability to mine the data and correlate with existing data and knowledge will play a critical role in generating valuable insights from the data. In addition to data integration, validation, and sharing to facilitate cross-study analysis, it is important to assimilate, mine and filter relevant data and facilitate expert reviews on multiple channels of information.

**Figure 9.**
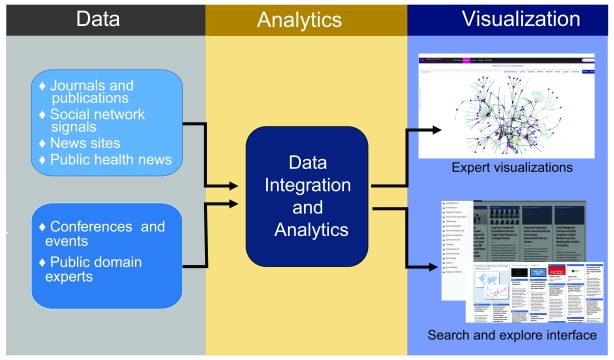
Concepts of an intelligent, knowledge mining and visualization platform for systems toxicology.

The platform outlined in this paper envisages to integrate such an intelligent system for knowledge mining, visualization and learning from multiple datasets, as illustrated schematically in Figure 9.

In the future, the system will support data accumulation and assimilation from multiple sources beyond experimental data and publications, automatically integrate and mine the multi-dimensional data through machine-learning and text-mining algorithms to identify and visualize scientifically relevant information and nominate experts for reviews.

We envision a future where our data platform, closely coupled with such an intelligent knowledge-mining system with cloud-based visualization and a search interface will power this systems toxicology platform.

## Conclusions

Our reported data management method employs the latest standards of data sharing and reproducible research. The data and methods curated and prepared in ISA-Tab format, fit for review by scientists, are stored in a database that can be accessed via a search portal on the website. As we continue developing the platform, we will also take into account how to make datasets more FAIR, namely by adding schemata to the datasets as recently recommended in a Nature Genetics editorial ^56^. The portal allows browsing data and information related to study design, materials and methods, and key results by either study or mechanism (e.g., inflammation or oxidative stress). A future update is planned to provide analytics for scientists and regulators to explore the data.

The platform is also intended to foster collaboration in the development of assays for the assessment of the future potential MRTPs. Therefore, protocols are described in detail, and future updates of the platform will allow versioning and commenting of protocols used for data generation and/or data analysis.

Given the successful development of the initial infrastructure, our goal is to grow this initiative into a public repository for 21 ^st^-century preclinical systems toxicology assessment data for MRTPs following the Morven Core Principles ^14^, as described above.

We hope that the infrastructure reported in this paper sparks interest and encourages other industries and institutions producing data relevant to MRTPs to submit their data to this repository for the benefit of the entire community.

## Data availability

The data referenced by this article are under copyright with the following copyright statement: Copyright: © 2017 Boué S et al.

The data can be browsed and downloaded from the website www.intervals.science.

## Supplementary material

**Supplementary File 1:** List of ontologies used in the ISA-Tab files describing *in vivo* and *in vitro* studies.
