# Supplementary material for: Supporting evidence-based analysis for modified risk tobacco products through a toxicology data-sharing infrastructure
Source: F1000Res. 2017 Sep 5;6:12. Originally published 2017 Jan 5. [Version 2] doi: 10.12688/f1000research.10493.2 (PMC5657032; doi:10.12688/f1000research.10493.2)
Supplement: Peer review history

# Peer review: Referee response for version 2

**DOI:** 10.5256/f1000research.13594.r25698

**Published:** 2017-10-24

**Authors:** Winston A. Hide, Sarah Morgan

**Affiliations:** 1 Department of Neuroscience, Sheffield Institute of Translational Neuroscience, University of Sheffield, Sheffield, MS, UK; 2 Sheffield Institute for Translational Neuroscience, University of Sheffield, Sheffield, UK

The authors have addressed the majority of concerns.

We have read this submission. We believe that we have an appropriate level of expertise to confirm that it is of an acceptable scientific standard.

# Peer review: Referee response for version 1

**DOI:** 10.5256/f1000research.11308.r24812

**Published:** 2017-08-14

**Authors:** Katharine Briggs

**Affiliations:** 1 Lhasa Limited, Granary Wharf House, 2 Canal Wharf, Leeds, UK

Here are my comments on this article:

- Page 3: “ *Concerns on reproducibility of science have lead to recent calls for a shift to better practices*” – correct to ‘led’
- Page 3: “ *Building and quantifying AOPs requires multiscale integration of all available and relevant -omics datasets,*” – drop the ‘-omics’
- Page 7: *“The table highlights sources of information on in vivo chemical inhalation and individual in vitro chemical toxicity. The type of data available and user accessibility (e.g., open source vs licensing) have also been highlighted.”* – User accessibility is not provided in all cases so I suggest adding ‘where known’ to caption
- Page 8: *“and allapplications need to employ sound, reproducible scientific bases and good practices”* – please correct to ‘and all applications’ and ‘basis’
- Page 9: *“Experiments are planned and protocols as well as metadata are recorded for each of the data entry and curated in ISA-Tab files.”* – Consider rewording of the Figure 2 caption as meaning is unclear
- Page 9: *“…on organotypic acute human buccal or nasal tissue cultures (i.e., the organotypic buccal and nasal datasets, respectively) …three dimensional (3-D) organotypic buccal and nasal epithelial culture models …in vitro human 3-D buccal or nasal epithelial culture …in vitro human 3-D buccal or nasal epithelial culture”* – Consider rewording point 2) as text is repetitive
- Page 14: *“Although this will include formal checks of files’ correctness with respect to the ISA-Tab standard (as already performed, e.g., by ISACreator), equipment data, log files, already-available databases, and computational infrastructure will be interfaced to provide the needed information at least partially.”* – Consider rewording as meaning is unclear
- Page 15: *“researchers need to focus on the biological small data”* – What is meant by biological small data?
- Conclusion: *“the platform will allow versioning and commenting of protocols”* – clarification is needed on the term protocol; is this study protocols or data analysis protocols?
- Page 9: *“Figure 9. Concepts of an intelligent, knowledge mining and visualization platform for systems toxicology.”* – Clarification needed on the link between Figure 9 and the contents of this article
- Page 18: Reference Source – the link to reference 14 is not working
- Page 19: *“* *Meyer P, Alexopoulos LG, Bonk T, et al.: Verification of systems biology research in the age of collaborative competition. Nat Biotechnol. 2011; 29(9): 811–5.”* – Reference 39 is a duplicate of reference 18

 

 In the Supplementary data:

- The MESH & LOINC entries are missing a description
- *“A vocabulary for clinical care, translational and basic research, and public information and administrative activities.”* - … and basic research, public information and administrative activities.
- *“Mainly because of the emerging need of systems toxicology to controlled vocabularies and also the lack of suitable ontologies for this domain, the CSEO prepares the ground for integrative systems-based research in the exposure science.”* - …emerging need of systems toxicology for controlled vocabularies… in exposure science.
- *“Metrical units for use in conjunction with PATO” *– What is PATO?
- *“  A structured classification of chemical compounds of biological relevance.” – *Remove space before A
- *“The set of standardized ontologies used to define the domain-specific knowledge are found in Table 1” *– Where is the Table 1 referred to?
- “Ontology developed to harmonize the toxicology datasets from the pharmaceutical industry made available in the eTox project and allow for comparative data-mining across multiple databases to test hypotheses” - …harmonize the nonclinical toxicology datasets… and allows comparative… multiple datasets to test hypotheses

I have read this submission. I believe that I have an appropriate level of expertise to confirm that it is of an acceptable scientific standard.

# Response

**Published:** 2017-08-30

**Authors:** Stephanie Boue

**Affiliations:** Philip Morris, Switzerland

- Page 3: “ *Concerns on reproducibility of science have lead to recent calls for a shift to better practices*” – correct to ‘led’
  - Author’s response: Thanks for the comment, this has been corrected
- Page 3: “ *Building and quantifying AOPs requires multiscale integration of all available and relevant -omics datasets,*” – drop the ‘-omics’
  - Author’s response: Thanks for the comment, this has been corrected
- Page 7: *“The table highlights sources of information on in vivo chemical inhalation and individual in vitro chemical toxicity. The type of data available and user accessibility (e.g., open source vs licensing) have also been highlighted.”* – User accessibility is not provided in all cases so I suggest adding ‘where known’ to caption
  - Author’s response: Thanks for the comment, this has been added
- Page 8: *“and allapplications need to employ sound, reproducible scientific bases and good practices”* – please correct to ‘and all applications’ and ‘basis’
  - Author’s response: Thanks for the comment. The original word seems fine, we will make sure the justification in the final document doesn’t impair reading.
- Page 9: *“Experiments are planned and protocols as well as metadata are recorded for each of the data entry and curated in ISA-Tab files.”* – Consider rewording of the Figure 2 caption as meaning is unclear
  - Author’s response: Thanks for the comment. The sentence has been modified and hopefully clearer now.
- Page 9: *“…on organotypic acute human buccal or nasal tissue cultures (i.e., the organotypic buccal and nasal datasets, respectively) …three dimensional (3-D) organotypic buccal and nasal epithelial culture models …in vitro human 3-D buccal or nasal epithelial culture …in vitro human 3-D buccal or nasal epithelial culture”* – Consider rewording point 2) as text is repetitive
  - Author’s response: Thanks for the comment. The paragraph has been modified.
- Page 14: *“Although this will include formal checks of files’ correctness with respect to the ISA-Tab standard (as already performed, e.g., by ISACreator), equipment data, log files, already-available databases, and computational infrastructure will be interfaced to provide the needed information at least partially.”* – Consider rewording as meaning is unclear
  - Author’s response: Thanks for the comment. The paragraph has been modified.
- Page 15: *“researchers need to focus on the biological small data”* – What is meant by biological small data?
  - Author’s response: Thanks for the comment. We have tried to explain this concept, namely that on itself the biological data may seem insignificant, but when analyzed in the right context and with the right tools contributes meaningful information.
- Conclusion: *“the platform will allow versioning and commenting of protocols”* – clarification is needed on the term protocol; is this study protocols or data analysis protocols?
  - Author’s response: Thanks for the comment. Any protocol on the platform, from study setup to data generation and analysis will be versioned and open for commenting. We explicated this in the text.
- Page 9: *“Figure 9. Concepts of an intelligent, knowledge mining and visualization platform for systems toxicology.”*– Clarification needed on the link between Figure 9 and the contents of this article
  - Author’s response: The authors thank the reviewer for the point raised. The section related to Fig.9 lays out the future application of the data platform outlined in this phase, wherein the ability to mine the data and correlate with existing data and knowledge will play a critical role in generating valuable insights from the data. Thus, the section outlined our vision and plan to further enhance the data platform with an intelligent system which automatically integrates and mines the multi-dimensional data through machine-learning and text-mining algorithms to identify and visualize scientifically relevant information and nominate experts for reviews. The relevant section has been updated to clarify and elucidate the role of the intelligent system, built on top of the data platform, as depicted in Fig. 9.
- Page 18: Reference Source – the link to reference 14 is not working
  - Author’s response: Thanks for the comment. The link has been changed.
- Page 19: *“Meyer P, Alexopoulos LG, Bonk T, et al.: Verification of systems biology research in the age of collaborative competition. Nat Biotechnol. 2011; 29(9): 811–5.”* – Reference 39 is a duplicate of reference 18
  - Author’s response: Thanks for the comment. The duplicated reference has been removed.

 

 In the Supplementary data:

- The MESH & LOINC entries are missing a description
  - Author’s response: Thanks for the comment. A description has been added.
- *“A vocabulary for clinical care, translational and basic research, and public information and administrative activities.”* - … and basic research, public information and administrative activities.
- *“Mainly because of the emerging need of systems toxicology to controlled vocabularies and also the lack of suitable ontologies for this domain, the CSEO prepares the ground for integrative systems-based research in the exposure science.”* - …emerging need of systems toxicology for controlled vocabularies… in exposure science.
  - Author’s response: Thanks for the comment, this has been corrected.
- *“Metrical units for use in conjunction with PATO” *– What is PATO?
  - Author’s response: Thanks for the comment, the acronym was explained (Phenotype And Trait Ontology)
- *“  A structured classification of chemical compounds of biological relevance.” – *Remove space before A
  - Author’s response: Thanks for the comment, this has been corrected.
- *“The set of standardized ontologies used to define the domain-specific knowledge are found in Table 1” *– Where is the Table 1 referred to?
  - Author’s response: Thanks for the comment, this has been corrected.
- “Ontology developed to harmonize the toxicology datasets from the pharmaceutical industry made available in the eTox project and allow for comparative data-mining across multiple databases to test hypotheses” - …harmonize the nonclinical toxicology datasets… and allows comparative… multiple datasets to test hypotheses
  - Author’s response: Thanks for the comment, this has been corrected.

# Peer review: Referee response for version 1

**DOI:** 10.5256/f1000research.11308.r21168

**Published:** 2017-03-22

**Authors:** Winston A. Hide, Sarah Morgan

**Affiliations:** 1 Department of Neuroscience, Sheffield Institute of Translational Neuroscience, University of Sheffield, Sheffield, MS, UK; 2 Sheffield Institute for Translational Neuroscience, University of Sheffield, Sheffield, UK

Boué *et al.* have built a toxicology data-sharing infrastructure for assessing modified risk tobacco products (MRTPs). They have described used best practice guidelines

  

1. On the INTERVALS website, the authors have exhaustively described the methods of each experiment under the “studies” section. At the end of the page is a results section where a huge amount of data is presented under many tabs which need to be selected in order to see the data. While the description of the methods is immensely important, the focus should surely be around the individual chemical being analysed or at least the results of the study. For example, there is no easy way to observe all the results for THS2.2. I would suggest either removing the use of tabs or, better yet, moving all results to their own section on the website (without the use of tabs) under the name of the MRTP so that all the results are easy to browse with links back to the methods used. This would enable the user to easily examine their MRTP of interest.
2. Again on the “data” section, where the data is available to download, the authors have focused filtering by methods rather than filtering by MRTP. If someone wants to assess the potential of THS2.2, they would want to view all the results for this chemical. Secondly, if possible, a feature enabling the user to download multiple datasets (instead of clicking on 37 links for THS2.2) would be incredibly useful.
3. The authors have highlighted the importance of the searchability of the data yet they have constrained their own search engine by the terms listed on the “data” section. Currently, you cannot search for any information contained within the metadata. The authors should think about including this in the future.
4. The use of “pMRTP” is very confusing and badly named. Especially given that the website glossary definition of it is “See MRTP definition” which mentions nothing of pMRTP. I understand that the original publication on “pMRTP” uses this name but it’s very unclear if this is a chemical/method of heating. If named such, is it an accepted MRTP? As far as I am aware, the FDA has not approved any MRTPs. On the website, the “pMRTP - 7-month systems toxicology inhalation / cessation study in C57BL6 mice” study also fails to mention what it is. A clearer definition is needed on all pages of the website and for the first mention in the paper.
5. The authors have turned their ISA-TAB files to excel format. This is prone to excel-specific errors, for example, turning number entries into dates. Why not use the actual ISA-TAB format?
6. Page 10:
  “Therefore, herein, we propose a new ISA-Tab scheme that follows the data-production workflow and combines data production and documentation into a single step by the researcher, who is the expert on the dataset and experimental parameters.”
  The authors have proposed this solution to the fact that creating ISA-TAB files is “complex, time-consuming, and error-prone”. This doesn’t appear to solve the issue fully. How do the authors expect to convince researchers to fill out ISA-TAB files as the experiment is ongoing? ISA-TAB files are complex and each researcher needs training on how to complete these correctly. While the authors have described how to complete ISA-TAB files, it is not easy to perform.
  They have also suggested ontologies to use but not the standardised terms to use within these ontologies. Each researcher might use different terms. Are the authors going to check over all the ISA-TAB files themselves? When the authors mention “extensions to the ISA-TAB templates”, are they going to provide a web template which creates the ISA-TAB files for the user? Additionally, the suggested ontologies should also be listed on the website.
  Page 14:
  “The first step in this direction is the development of a data input and management application consisting of several validated web forms and lists with filtering/searching features connected to the uploaded datasets”.
  Please describe how these were validated. Where are the web forms located on the website? Are these for premade ISA-TAB files or for creating the ISA-TAB files? Please explain more.
7. Who can add data to the website? There is no option for new members to join, or upload data, will this change in the future?
8. There are no publication references on the “data” section of the website. If possible, it would be better to include this.
9. There are no obvious explanations of the headers within the downloadable data yet a number of abbreviations are used. Please think about changing this as soon as possible.

We have read this submission. We believe that we have an appropriate level of expertise to confirm that it is of an acceptable scientific standard, however we have significant reservations, as outlined above.

# Response

**Published:** 2017-08-30

**Authors:** Stephanie Boue

**Affiliations:** Philip Morris, Switzerland

*1. On the INTERVALS website, the authors have exhaustively described the methods of each experiment under the “studies” section. At the end of the page is a results section where a huge amount of data is presented under many tabs which need to be selected in order to see the data. While the description of the methods is immensely important, the focus should surely be around the individual chemical being analysed or at least the results of the study. For example, there is no easy way to observe all the results for THS2.2. I would suggest either removing the use of tabs or, better yet, moving all results to their own section on the website (without the use of tabs) under the name of the MRTP so that all the results are easy to browse with links back to the methods used. This would enable the user to easily examine their MRTP of interest.*

Author’s response: Thanks for the comment, which is very relevant, and would become even more so as the platform grows and the number of studies increases. Since the publication of the first version of this manuscript, we have updated the prototype platform and are building a new platform that will enable easier access to all relevant pieces of information and should replace the current prototype, at the same URL, by the end of the year.

A major update is the focus on the test item that has been added to the studies section. It is now easier to find all studies related to a specific test item. Results are, in the prototype, still in tabs, but we have taken the comment into account for the building of the new platform and will add tags and facet search to ease retrieval of results and protocols of interest.

*2. Again on the “data” section, where the data is available to download, the authors have focused filtering by methods rather than filtering by MRTP. If someone wants to assess the potential of THS2.2, they would want to view all the results for this chemical. Secondly, if possible, a feature enabling the user to download multiple datasets (instead of clicking on 37 links for THS2.2) would be incredibly useful.*

Author’s response: Thanks for the comment. Again, this change will be addressed in the new platform. We are also working on the addition of analytical tools directly on the platform to ease data visualization and analysis.

*3. The authors have highlighted the importance of the searchability of the data yet they have constrained their own search engine by the terms listed on the “data” section. Currently, you cannot search for any information contained within the metadata. The authors should think about including this in the future.*

Author’s response: Thanks for the very valuable comment. Again, this change will be addressed in the new platform. In addition to the facets that will be available for easy filtering, the user will have the option to search for text in a number of fields of the datasets.

*4. The use of “pMRTP” is very confusing and badly named. Especially given that the website glossary definition of it is “See MRTP definition” which mentions nothing of pMRTP. I understand that the original publication on “pMRTP” uses this name but it’s very unclear if this is a chemical/method of heating. If named such, is it an accepted MRTP? As far as I am aware, the FDA has not approved any MRTPs. On the website, the “pMRTP - 7-month systems toxicology inhalation / cessation study in C57BL6 mice” study also fails to mention what it is. A clearer definition is needed on all pages of the website and for the first mention in the paper.*

Author’s response: Thanks for the comment. MRTP stands for modified risk tobacco product, as defined in the abstract and introduction of the publication. It is the term chosen by the FDA to refer to products ‘sold or distributed to reduce the harm or risk of tobacco-related disease associated with commercially marketed tobacco products’. The study mentioned pMRTP where the p stands for prototype, as at the time when it was assessed, the product was not sold nor distributed.

Moreover, we have updated the website to refer to reduced risk products (RRPs) instead as this is the term we use to refer to products that present, are likely to present, or have the potential to present less risk of harm to smokers who switch to these products versus continued smoking. We have a range of RRPs in various stages of development, scientific assessment and commercialization. Because our RRPs do not burn tobacco, they produce far lower quantities of harmful and potentially harmful compounds than found in cigarette smoke. We have included the definition in the glossary and ensured that the definition is easily accessible when the term is encountered initially on the website (pop-up with definition on mouse-over).

*5. The authors have turned their ISA-TAB files to excel format. This is prone to excel-specific errors, for example, turning number entries into dates. Why not use the actual ISA-TAB format?*

Author’s response: Thanks for the comment. We have decided to split the ISA-TAB files by endpoint and stage of the study (starting material, data production, data processing…) to allow easier recording of data and metadata as the experiment is performed by the scientists and technicians in charge. The splitting of the ISA-TAB files by endpoint also allows easier interpretation and reuse of data by others, but it results in a large number of tabs, which would all be separated files when using the CSV format as proposed in the ISA-TAB standard. Using the multiple tab format of Excel allows for the grouping of all tabs belonging to a specific endpoint and, in this way, easier data handling for the end user. Additionally, by the careful preparation of the data files in Excel, we were able to avoid all Excel-specific errors occurring during the automatic conversion into the XLS format during opening CSV files in Excel, a problem correctly stressed by the reviewer. Since Excel is very often used in the domain, we hope to help avoiding these errors by directly providing XLS file, which can be easily concerted to CSV files if needed.

*6. Page 10:*

*“Therefore, herein, we propose a new ISA-Tab scheme that follows the data-production workflow and combines data production and documentation into a single step by the researcher, who is the expert on the dataset and experimental parameters.”*

*The authors have proposed this solution to the fact that creating ISA-TAB files is “complex, time-consuming, and error-prone”. This doesn’t appear to solve the issue fully. How do the authors expect to convince researchers to fill out ISA-TAB files as the experiment is ongoing? ISA-TAB files are complex and each researcher needs training on how to complete these correctly. While the authors have described how to complete ISA-TAB files, it is not easy to perform.*

*They have also suggested ontologies to use but not the standardised terms to use within these ontologies. Each researcher might use different terms. Are the authors going to check over all the ISA-TAB files themselves? When the authors mention “extensions to the ISA-TAB templates”, are they going to provide a web template which creates the ISA-TAB files for the user? Additionally, the suggested ontologies should also be listed on the website.*

*Page 14:*

*“The first step in this direction is the development of a data input and management application consisting of several validated web forms and lists with filtering/searching features connected to the uploaded datasets”.*

*Please describe how these were validated. Where are the web forms located on the website? Are these for premade ISA-TAB files or for creating the ISA-TAB files? Please explain more.*

Author’s response: We apologize that the wording here was not clear enough. The application only facilitates the upload of pre-generated ISA-TAB files and the assignment of facets for the search/browse features. This is now clearly described in the manuscript. We also removed the somewhat misleading “validated” in front of “web forms”.

The list of ontologies we recommend can be found in this publication as supplementary Excel file 1 and will be available from the platform once the data upload will be available for non-PMI users.

*7. Who can add data to the website? There is no option for new members to join, or upload data, will this change in the future?*

Author’s response: Thanks for the very valuable comment. This publication describes indeed the prototype and ideas important for the sharing of data. We are currently building a new platform that will allow scientists who experimented on potential RRPs to share their protocols, their data and results. The platform with necessary features should be launched by the end of the year. 

*8. There are no publication references on the “data” section of the website. If possible, it would be better to include this.*

Author’s response: Thanks for the comment. It is not fully clear which references should be added to the data section of the website. References important for specific studies can be found in the study pages. Those important for the protocols are found in the respective material and methods sections.

We acknowledge it would be great to have a single ID associated to datasets so they can be cited on the platform and are evaluating this possibility.

*9. There are no obvious explanations of the headers within the downloadable data yet a number of abbreviations are used. Please think about changing this as soon as possible.*

Author’s response: Thanks for the comment. We have, to some extent, reworked the files so they are more self-explanatory. Schema files and/or readme files will be added consistently to the dataset archives by the end of the year (on the new platform).
